# Exploring the Ecology of Bifidobacteria and Their Genetic Adaptation to the Mammalian Gut

**DOI:** 10.3390/microorganisms9010008

**Published:** 2020-12-22

**Authors:** Sabrina Duranti, Giulia Longhi, Marco Ventura, Douwe van Sinderen, Francesca Turroni

**Affiliations:** 1 Laboratory of Probiogenomics, Department of Chemistry, Life Sciences and Environmental Sustainability, University of Parma, Parco Area delle Scienze 11a, 43124 Parma, Italy; sabrina.duranti@unipr.it (S.D.); giulia.longhi@unipr.it (G.L.); marco.ventura@unipr.it (M.V.); 2 GenProbio srl, 43124 Parma, Italy; 3 Microbiome Research Hub, University of Parma, 43124 Parma, Italy; 4 APC Microbiome Institute and School of Microbiology, Bioscience Institute, National University of Ireland, H91 TK33 Cork, Ireland; d.vansinderen@ucc.ie

**Keywords:** *Bifidobacterium*, microbial ecology, microbe–host interaction, microbe–microbe interaction

## Abstract

The mammalian gut is densely inhabited by microorganisms that have coevolved with their host. Amongst these latter microorganisms, bifidobacteria represent a key model to study host–microbe interaction within the mammalian gut. Remarkably, bifidobacteria naturally occur in a range of ecological niches that are either directly or indirectly connected to the animal gastrointestinal tract. They constitute one of the dominant bacterial members of the intestinal microbiota and are among the first colonizers of the mammalian gut. Notably, the presence of bifidobacteria in the gut has been associated with several health-promoting activities. In this review, we aim to provide an overview of current knowledge on the genetic diversity and ecology of bifidobacteria. Furthermore, we will discuss how this important group of gut bacteria is able to colonize and survive in the mammalian gut, so as to facilitate host interactions.

## 1. History and Taxonomy of Bifidobacteria

The Actinobacteria phylum represents one of the most numerous and heterogeneous groups of microorganisms present in nature [1,2]. These Gram-positive bacteria are characterized by a high GC genome content ranging from 51% to more than 70%, and exhibit different morphologies, including unicellular cocci or rods, and complex multicellular consortia [1,2]. Furthermore, these bacteria are able to produce bioactive natural compounds and these features are reflected in their ability to adapt to several quite distinct ecosystems such as various terrestrial and aquatic environments, as well as the bodies of mammals and birds [1,2]. In fact, this phylum includes pathogens (e.g., *Mycobacterium* spp., *Nocardia* spp., *Tropheryma* spp., *Corynebacterium* spp., and *Propionibacterium* spp.), soil inhabitants such as *Streptomyces* spp., plant commensals (e.g., *Leifsonia* spp.), nitrogen-fixing symbionts (*Frankia*), and human gut inhabitants (*Bifidobacterium* spp.) [1,2].

**Figure 1:**
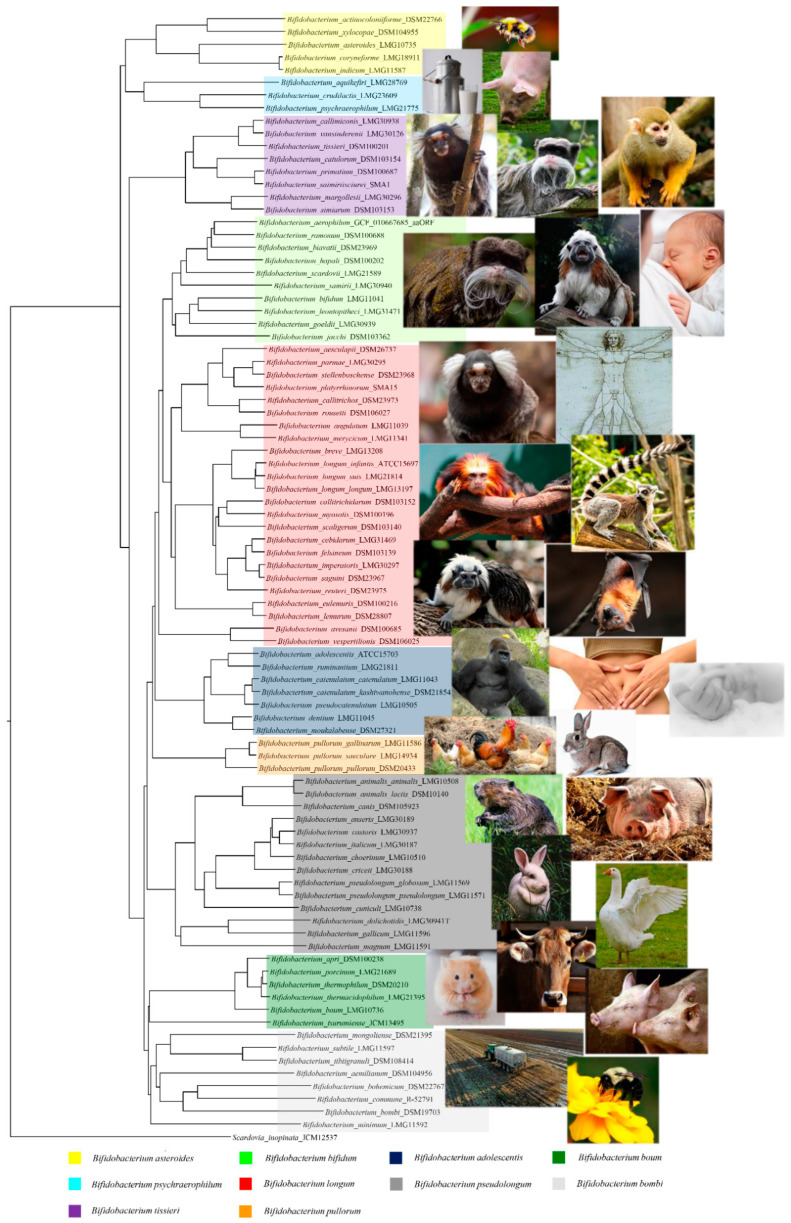
Phylogenetic tree of the *Bifidobacterium* genus based on the concatenation of 191 core amino acid sequence genes. The core genes-based tree shows the subdivision of the 10 phylogenetic groups of the *Bifidobacterium* genus represented with different colors. The phylogenetic tree was built by the neighbor-joining method with corresponding sequences of *Scardovia inopinata* JCM 12,537 being employed as outgroup. Bootstrap percentages above 50 are shown at node points, based on 100 replicates of the phylogenetic tree. The ecological origins of the various phylogenetic groups are highlighted beside the phylogenetic tree.

The genus *Bifidobacterium* belongs to the *Bifidobacteriaceae* family, Bifidobacteriales order, and these bacteria were isolated, for the first time, from feces of a breast-fed infant by Tissier in 1899 [3]. They represent nonmotile, anaerobic, nonsporulating, saccharolytic bacteria with a bifid or multiple-branching rod morphology. Currently, the genus *Bifidobacterium* comprises 94 taxa, representing 82 species and 12 subspecies [4,5,6,7,8,9,10,11,12] (Table 1). In recent years, the phylogeny of the *Bifidobacterium* genus has been explored using different methods based on the sequencing of the 16S rRNA gene, by means of a multilocus approach, or the sequencing of several housekeeping genes (i.e., *clpC*, *dnaJ*, *rpoC*, *xpf*, *dnaB*, and *purF*) [13,14]. A comparative genomics analysis based on all 88 sequenced bifidobacterial type strains revealed the presence of 191 *Bifidobacterium*-specific clusters of orthologous genes (COGs) shared by these genomes, called the bifidobacterial core-genome [15]. Notably, the phylogenetic tree constructed by amino acid concatenation of these 191 bifidobacterial core-genome genes revealed the existence of 10 different phylogenetic groups, encompassing *Bifidobacterium adolescentis*, *Bifidobacterium boum*, *Bifidobacterium pullorum*, *Bifidobacterium asteroides*, *Bifidobacterium longum*, *Bifidobacterium psychraerophilum*, *Bifidobacterium bifidum*, *Bifidobacterium pseudolongum*, *Bifidobacterium bombi*, and *Bifidobacterium tissieri* groups [15]. These groups partially correlate with the ecological niches from which the representative species were isolated. For example, members of the *B. tissieri* group are common inhabitants of the microbiota of tamarin and those of the *B. pullorum* group are characteristic of birds. According to this, members of the *B. adolescentis* group (*Bifidobacterium catenulatum*, *Bifidobacterium pseudocatenulatum*, and *B. adolescentis* strains), the *B. longum* group (*Bifidobacterium breve* and *B. longum* strains), the *B. pseudolongum* group (especially *Bifidobacterium animalis* subsp. *lactis* strains), and the *B. bifidum* group (*B. bifidum* strains) are typical colonizers of the human intestinal tract or are commercially exploited as probiotic strains (Figure 1).

## 2. Ecology of Bifidobacteria

Bifidobacteria also naturally occur in the gastrointestinal tract (GIT) of animals, such as nonhuman mammals, insects, and birds [5,6,7,8,9,10,11], while they have also been isolated from human blood [51], sewage [42], the oral cavity [55], and fermented milk [15]. In this context, it has been demonstrated that the ability of bifidobacteria to adapt to specific environments is species-dependent [4]. Until recently, scientific studies revealed that *B. longum*, *B. adolescentis*, *B. pseudolongum*, and *B. bifidum* species possess a cosmopolitan lifestyle [4], whereas other bifidobacterial species appear to be adapted to the GIT of particular animals (e.g., *Bifidobacterium cuniculi* for rabbits, *Bifidobacterium angulatum* for cows, and *Bifidobacterium gallinarum* for chickens) or the human gut (e.g., *B. breve* and *B. longum* species) [4,12]. However, recent ecological studies, based on Internally Transcribed Spacer (ITS) profiling, have revealed that the distribution of *Bifidobacterium* species is not host-specific [57,58]. For example, the *B. breve* species, which until that point had only been associated with the human gut, was shown to be present also in domesticated animals [57]. Furthermore, particular species, such as *Bifidobacterium actinocoloniiforme*, *B. asteroides*, *Bifidobacterium bohemicum*, *B. bombi*, and *Bifidobacterium indicum,* which were previously thought to be highly specialized to colonize the insect gut, were shown to be widely distributed among various mammalian hosts [58]. Notably, the distribution of bifidobacterial species in different ecological niches reinforces the idea that anthropogenic influences may have promoted such apparent horizontal transmission events.

The *Bifidobacterium* genus is one of the most abundant bacterial genera present in the human gut during the early stages of life [59,60,61] and these microorganisms are reported to be among the first bacterial colonizers of the newborn’s GIT [62]. It has been demonstrated that bifidobacteria may engage in vertical transmission that occurs between a mother and her newborn during birth and possibly through subsequent breastfeeding [63,64]. This fascinating phenomenon not only occurs in human beings [65] but also in other mammalian species [58]. In this context, some studies have shown how taxonomic classification of bifidobacteria present in the mother’s microbiota strongly correlates with that of the infant [66,67]. In particular, a study based on ITS-profiling and shotgun-metagenomics approaches has led to the identification of the species shared between a mother and her child [68]. In this study, the microbiota of a mother’s fecal and milk samples were assayed together with corresponding infant fecal samples collected at different time points. These analyses demonstrated that in some cases, identical bifidobacterial strains are shared in both mother’s and baby’s gut microbiota [68]. A *B. breve* strain and *B. longum* subsp. *longum* isolate were seen to be among the protagonists of vertical transmission from mother to child, being found both in the newborn’s meconium and in the fecal samples of the child for up to 90 days [68]. Several species of this genus are believed to have undergone specific genetic and metabolic adaptations in order to facilitate colonization of the infant gut, for example, the ability of certain bifidobacterial species and strains to metabolize specific oligosaccharides present in human milk [69]. Specifically, bifidobacterial species that are prevalent in the gut of infants include *B. breve*, *B. longum* subsp. *infantis*, *B. longum* subsp. *longum*, *B. pseudocatenulatum*, and *B. bifidum* [62], whereas *B. adolescentis*, *B. catenulatum*, *B. pseudocatenulatum*, and *B. longum* subsp. *Longum* [70,71] are commonly occurring species in the adult intestine. In this context, it is not fully correct to consider the use of fecal material as a representation of the entire intestinal microbiota. In fact, the fecal microbiota consists not only of mucosal adherent members of the human GIT microbiota but also of transient bacteria derived from the diet or other environmental microbial contaminations [72]. Specifically, only a small number of bifidobacterial species (i.e., *B. longum*, *B. adolescentis*, *B. breve*, *B. pseudocatenulatum*, and *B. pseudolongum*) seem to be dominant in the examined biopsies, whereas certain other bifidobacterial species are restricted to a specific ecological niche (e.g., *B. bifidum* and *B. pseudolongum*) [73]. Analyses not only of human intestinal mucosal but also of fecal samples have shown that bifidobacterial distribution changes within ages, with a remarkable conservation in terms of species and strains in adults and children [73]. Furthermore, little is known about the diversity of bifidobacterial populations occurring between individuals and between different compartments of the GIT within the same individual [73].

The presence of bifidobacteria in the GIT has been associated with various health benefits, including the development of the immune system, protection against pathogens mediated through the process of competitive exclusion, and/or the production of metabolites such as short-chain fatty acids (SCFA) and vitamins [59,62,70,74]. Indeed, human-residential bifidobacteria (HRB) are also capable of producing folate, also known as vitamin B9 or B11, which is required for an efficient DNA replication, DNA repair/methylation, and synthesis of nucleotides, vitamins, and certain amino acids [75,76]. For these reasons, several bifidobacterial strains/species are used as active ingredients in a variety of so-called functional foods due to their perceived health-promoting or probiotic properties [2]. In this context, probiotic bifidobacterial strains belonging to *B. longum* and *Bifidobacterium animalis* subsp. *lactis* species are usually added to yogurt, other fermented milks, and, more recently, to cheese, which are the most popular probiotic foodstuffs at the moment [77,78]. Moreover, clinical studies have demonstrated that *B. animalis* ssp. *lactis* Bb-12, administered as probiotic adjunctive therapy, have beneficial effects in the case of infectious diarrhea caused by viruses or bacteria [79,80], decreasing the frequency or shortening the duration of the infection and increasing immune responses [81].

## 3. Bifidobacteria and Their Genetic Adaptation to the Human Gut

In silico analysis of bifidobacterial genomes has uncovered molecular evidence illustrating how particular members of this genus have adapted to the human intestine. In particular, analysis of these data identified a large arsenal of genes encoding enzymes involved in the degradation of complex carbohydrates derived from the (mother of the) host (e.g., mucin and human milk oligosaccharides) or from the diet (e.g., starch, galactan, (arabino)xylan) [69,82,83]. Specifically, these carbohydrates cannot be digested by host enzymes or metabolized by most microorganisms of the gut microbiota, yet can be utilized by certain gut commensals, which may include bifidobacteria [82,83]. Clear examples of bifidobacteria that encode enzymes for host glycan degradation and utilization are *B. bifidum* PRL2010 and *B. longum* subsp. *infantis* ATCC15697 [69,82]. In this context, genome analysis of *B. bifidum* PRL2010 revealed that this strain encodes various enzymes that are responsible for mucin degradation, including a 1,2-α-L-fucosidase, a 1,3/4-α-L-fucosidase, putative exo-α-sialidases, N-acetyl-β-hexosaminidases, β-galactosidases, and a putative cell wall-anchored endo-α-N-acetylgalactosaminidase [82]. Furthermore, comparative genomic analyses of several *B. bifidum* strains highlighted that the genetic repertoire involved in mucin breakdown is well conserved in members of this taxon [84]. Moreover, the genome of *B. longum* subsp. *infantis* ATCC15697 harbors a genetic locus encompassing genes that encode enzymes involved in Human Milk Oligosaccharides (HMOs) degradation, such as a fucosidase, sialidase, β-hexosaminidase, and β-galactosidase [69]. Recently, comparative genome analysis involving two strains of *B. longum* subsp. *infantis* (i.e., ATCC15697 and Bi-26) revealed that the metabolic enzymes involved in HMOs utilization appear to be conserved in this bifidobacterial taxon [85]. This comparative analysis was confirmed by metabolite analysis as well as transcriptomics assays suggesting that *B. longum* subsp. *infantis* ATCC15697 is able to consume various HMOs simultaneously, whereas *B. longum* subsp. *infantis* Bi-26 is adapted to internalize relatively short and predominantly fucosylated HMOs [85]. In conclusion, these findings show that even though HMOs consumption is characteristic of *B. longum* subsp. *Infantis*, there is strain-level variation concerning the ability of particular strains to metabolize structurally distinct elements from the HMOs pool.

Another significant example of genetic adaptation of bifidobacteria to the human gut is represented by the *B. adolescentis* species, which can utilize certain dietary polymeric carbohydrates, such as resistant starch [82,83]. The structurally related glycans starch, amylopectin, and pullulan have previously been demonstrated to be utilized by different bifidobaterial species, such as *B. breve* and *B. adolescentis*. In particular, O’Connell Motherway et al., analysing the genome of *B. breve* UCC2003, have characterized an extracellular bifunctional class II amylopullulanase, known as *apu*B, that is crucial for this species to metabolize starch [86]. Specifically, in order to evaluate the activity of this enzyme, the *B. breve* UCC2003 strain was manipulated to generate *B. breve* UCC2003-*apu*B mutant [86]. This study showed that the *apu*B-encoded enzyme is involved in the hydrolysis of extracellular starch or long-chain maltooligosaccharides to produce shorter maltooligosaccharides [86]. Also, various members of the *B. adolescentis* species possess a genetic repertoire predicted to be involved in the breakdown of starch and starch derivates [83,87,88]. In this context, pan-genome analysis of *B. adolescentis* showed that these enzymes, predicted to represent an α-glucosidase, amylase, pullulanase, and cyclomaltodextrinase, and assumed to be required for the degradation of starch and starch-like glycans, are present in all strains analyzed except for *B. adolescentis* 703B and *B. adolescentis* JCM15918 [83]. Consistent with this observation is the finding that these two strains do not exhibit any appreciable growth on starch [83]. Notably, none of the genomes of these *B. adolescentis* strains contain genes involved in the metabolism of host-derived glycans [83]. Altogether, these findings support the notion that there is a specific adaptation of *B. adolescentis* to the adult human gut, where starch represents a substantial glycan part of the diet. In fact, infant-associated *Bifidobacterium* species, such as *B. bifidum* and *B. longum* subsp. *infantis,* have evolved towards an ecological niche where host-polysaccharides such as mucin and HMOs are present in high abundance, whereas the adult-associated *B. adolescentis* taxon is adapted to colonize an environment where certain dietary plant-derived glycans are expected to be present.

## 4. Example of Host–Bifidobacteria and Microbe–Microbe Interactions in the Human Gut

Commensal gut bacteria have evolved specific strategies to establish interactions with the human host through various extracellular molecules, which include pili, capsular polysaccharides or exopolysaccharides, and serine protease inhibitors [4,87,89,90]. Recent studies have shown that also certain metabolic molecules such as tryptophan-derived metabolites can mediate host–microbe interactions [91,92]. It has been demonstrated that indole-3-lactic acid (ILA) is the main tryptophan-derived metabolite produced by bifidobacteria [93]. ILA is produced in large quantities by certain species of bifidobacteria, such as *B. longum* subsp. *longum*, *B. longum* subsp. *infantis*, *B. breve*, and *B. bifidum*, which are commonly isolated from human infant fecal samples [93]. The precise role played by this metabolite in infant development is not yet clear, but studies have reported that microbial tryptophan metabolites may have a positive effect on inflammatory responses [94] and neurological functions [95], while also maintaining intestinal and systemic homeostasis [96].

The genomes of most bifidobacteria encode two different types of pili, known as the sortase-dependent pili and type IVb or Tad (Tight adherence) pili that are involved in microbe–host interactions [89,97,98,99]. Specifically, the biosynthetic machinery for sortase-dependent pilus production is encoded by a genetic locus that includes a gene encoding a major pilin protein, one or two genes involved in the synthesis of ancillary pilin proteins, and a gene encoding a pilus-specific sortase, implicated in the assembly of pilus subunits [97]. In particular, comparison of genome sequences of various bifidobacterial species highlights the genetic diversity of sortase-dependent pilus-encoding loci within this genus. Specifically, the number of sortase-dependent pilus loci is different between species or even among strains of the same species. For example, the genome of *Bifidobacterium dentium* Bd1 is predicted to contain seven sortase-dependent pilus loci, that of *B. bifidum* PRL2010 harbors three putative sortase-dependent pilus loci, while the genomes of *B. animalis* subsp. *lactis* DSM10140, *B. adolescentis* ATCC15703, and *B. longum* subsp. *infantis* ATCC15697 revealed the predicted presence of just one such locus [97]. Current knowledge concerning the fimbriome of *B. bifidum* PRL2010 indicates that sortase-dependent pili play an important role in the establishment and colonization of this strain within the human gut [97,100,101]. In this context, Turroni et al. observed that pili produced by *B. bifidum* PRL2010 are able to induce high levels of TNF-α cytokines, and to reduce the expression of other proinflammatory cytokines (e.g., IL-12). These data support the notion that *B. bifidum* PRL2010, representing one of the first colonizers of the infant gut, is able to modulate the immune system of a newborn. This feature may thus have very important implications for the infant host, whose immature immune system may be dependent on specific stimulatory signals with long-lasting health consequences [98].

Another key example of extracellular structures produced by bifidobacterial species that are known to modulate the interaction with their hosts is represented by the Tad pili, which are conserved in all known bifidobacterial genomes [89,99]. The gene cluster of Tad pili is composed of three domains, including the machinery for pilus formation (i.e., assembly and localization domain, pilin proteins domains, processing domain). In the context of assembly and localization of these extracellular appendages, pilus localization is presumed to be determined by the product of *tad*Z, while ATPase and pilus assembly activities are encoded by *tad*B and *tad*G. Pilin proteins consist of a pre-pilin precursor, encoded by *flp*, and minor pilin precursors which are specified by *tad*E and *tad*F, while processing of these pilin precursors is performed by a prepilin peptidase, encoded by *tad*N, which is located outside of the *tad* locus [102]. Bifidobacterial Tad pili have been characterized in *B. breve* UCC2003 [99]. In particular, in vitro and in vivo studies revealed that *B. breve* UCC2003 Tad pili play a key role in gut colonization of the host [99]. In this context, in vivo experiments using a germ-free murine model revealed an increase in host intestinal cell proliferation after five days following the administration of *B. breve* UCC2003 [103]. Conversely, administration of a *B. breve* UCC2003 derivative carrying a mutation in the *tad*E gene to mice showed a significantly lower level of cell proliferation when compared to the control group (where mice were administered the wild-type *B. breve* strain) [103]. These data identified that the TadE pseudopilin promotes colonic epithelial cell proliferation and growth, confirming that Tad pili produced by *B. breve* UCC2003 are indeed involved in an important aspect of microbe–host interactions.

Another important factor involved in host–microbe interaction is the bifidobacterial serine protease inhibitor (serpin), which is a member of a superfamily of proteins involved in the regulation of certain protease-mediated processes [104]. Analysis of available bifidobacterial genomes revealed the presence of serpin-encoding genes in just a few human gut-derived bifidobacterial species, in particular *B. longum* subsp. *longum*, *B. longum* subsp. *Infantis*, and *B. breve* [105]. In contrast, the genomes of other human gut bifidobacterial species such as *B. adolescentis*, *B. catenulatum, B. bifidum*, and *B. gallicum* do not contain a serpin-encoding gene in their genomes, suggesting that these species are protected from serine proteases through an alternative way or rely on cross-protection offered by (bifido)bacterial species that are able to produce such serpins [105]. The release of serine proteases occurs as a result of intestinal inflammation caused by bacterial infections or damage to the intestinal tissue, typical of inflammatory bowel disease or ulcerative colitis. In this context, self-produced serpins help gut bacteria such as bifidobacteria to protect their extracellular proteins (e.g., pilins or glycan-hydrolyzing enzymes, see above) from degradation by these host-derived proteases, which may be advantageous for colonization and nutrient acquisition purposes [105,106].

An in vitro assay based on cells of *B. breve* 210B treated with different proteases highlighted that transcriptional activation of the serpin gene is guided by specific proteases [105]. In particular, analysis of transcriptional profiling of *B. breve* 210B cells treated with several proteases revealed that papain is able to stimulate upregulation of the serpin-encoding gene locus [105]. Furthermore, Alvarez-Martin et al. demonstrated that transcription of the serpin-encoding gene in *B. breve* UCC2003 is controlled by a two-component regulatory system (2CRS), known as SerRK [107]. Moreover, data derived from the analysis of *B. breve* UCC2003-serU mutant experiments highlighted that the SerRK signaling pathway includes a SerU-dependent autoregulatory mechanism [107]. SerU is one of the two genes belonging to the *ser2003* locus and encoding a serpin-like protein. This positive autoregulatory loop seems to ensure further production of SerU when the encountered proteolytic activity is higher than the provided serpin-mediated protease inhibition [107]. Bifidobacterial serpins, by the inhibition of human proteases, such as α-antitrypsin and human neutrophil elastase, are intended to reduce exaggerated serine protease activity, which could otherwise cause pathological damage to tissue [108].

In addition to pili and serpins, other key bifidobacterial extracellular structures that were shown to mediate the interaction with the host are exopolysaccharides (EPSs). The EPS consists of repeated mono or oligosaccharides units linked by glycosidic linkages that may result in a wide variety of structurally distinct macromolecules [109]. Several studies have revealed that surface-associated EPSs of bifidobacteria support gut colonization [110,111,112]. Furthermore, genomic analysis of 48 bifidobacterial species revealed that all type strains, except for *B. bifidum* species, contain at least one *eps* cluster [109]. Across assessed bifidobacterial (sub)species and based on common genetic organization of *eps* gene clusters, nine distinct *eps* clusters have been identified. In particular, the *eps*1 and *eps*2 clusters are found in bifidobacterial taxa commonly isolated from the human intestine, whereas the *eps*3 and *eps*4 clusters are identified in the genomes of bifidobacterial (sub)species commonly recovered from the gut of various animals [109]. Moreover, the cocultivation of different bifidobacterial strains in fecal media that simulate the human gut of adults as well as infants revealed the upregulation of the genes involved in EPS biosynthesis [109]. Transcriptional regulation of *eps* genes during growth of bifidobacterial in multi-association showed modulation of EPS biosynthesis by several bifidobacterial taxa when present in the same environment [109]. In recent years, interest in EPS producers has grown due to the key role that they appear to play in promoting human health [113]. Particularly, it has been demonstrated that *B. breve* UCC2003 produces an EPS which is capable of modulating immune responses and reducing the infection of a gut pathogen [114]. The genome of *B. breve* UCC2003 possesses two sets of adjacent oppositely oriented genes encoding two distinct EPSs, whose expression depends on the orientation of a single promoter. Therefore, only one of the two types of EPSs can be produced at any time [112]. Fanning et al. have demonstrated that splenocytes isolated from naïve mice stimulated with the EPS-producing *B. breve* UCC2003 strain (EPS+) evoked a lower expression of proinflammatory cytokines compared to two EPS-deficient (EPS−) *B. breve* UCC2003 strains. This finding was further confirmed by in vivo analysis on mice orally fed with EPS+ and EPS− strains, suggesting that strains producing surface EPS (EPS+) failed to elicit a strong immune response compared with EPS-deficient variants [114]. *B. longum* subsp. *longum* 35,624 is another interesting bifidobacterial producer of EPS which was shown to play a key role in modulating the host immune response. Comparative genomic analyses revealed the presence of a gene cluster (*eps*624) which encodes the EPS biosynthetic machinery, and which, despite its conserved genomic location, exhibits genetic diversity in different *B. longum* subsp. *longum* strains [115]. A study based on the coculture of human Peripheral Blood Mononuclear Cells (PBMCs) or Monocyte-derived Dendritic Cells (MDDCs) with *B. longum* subsp. *longum* 35,624 and EPS-negative mutant derivative demonstrated that the lack of EPS may increase proinflammatory cytokine secretion [116]. Similarly, administration of these strains to mice as a model of colitis underlined that the EPS-producing strains are able to prevent disease, while the EPS-deficient derivative does not show any protection against the development of colitis [116]. All together, these findings emphasize that the EPS of various bifidobacterial strains plays an important role in reducing the proinflammatory response.

Nowadays, much is known about microbe interactions with the human gut, also thanks to the great importance that bifidobacteria have gained as health-promoting bacteria. Given their wide ecological distribution, the challenge for the future is to deepen the knowledge of this field by studying other bifidobacterial mammalian strains/species as well.

## 5. Microbe–Microbe Cross-Feeding Activities Influence Intestinal Immune Homeostasis and Inflammatory Response

As mentioned above, bifidobacteria are microorganisms which are able to degrade certain complex carbohydrates that are indigestible to the host [74]. Carbohydrate metabolism by gut commensals leads to the production of SCFAs, of which acetate, propionate, and butyrate are most relevant in the context of the human gut. These SCFAs perform key functions such as increasing the absorption of calcium and magnesium, providing nutrients for the colonocytes, and stimulating the host immune system [117,118]. Among the SCFAs produced in the colon, butyrate represents a compound of high importance and impact for the gut. In fact, it is a source of energy for the intestinal mucosa cells, stimulating their replication [119]. Unable to directly synthesize butyrate, bifidobacteria resort to a mutual beneficial cross-feeding interaction with butyrogenic members of the intestinal microbiota such as *Faecalibacterium prausnitzii* [120]. In this context, bifidobacteria degrade complex carbohydrates producing acetate, which then becomes a source of energy for secondary degraders, which use acetate to generate butyrate. The cross-feeding interaction is mutually beneficial as both strains benefit from each other’s presence through the processing of metabolites favoring the coexistence in the same ecological niche. Bifidobacteria create numerous trophic interactions with each other and with other members of the gut microbiota. It has been demonstrated that cocultivation of *B. longum* NCC2705 and *Eubacterium rectale* ATCC 33,656 in a medium rich in arabinoxylo-oligosaccharides (AXOS) positively influences growth of both strains [121]. Specifically, *B. longum* NCC2705 possesses the enzymes capable of degrading AXOS, resulting in the release of xylose backbone (XOS) and acetate. The latter is used by *E. rectale* ATCC 33,656 to produce butyrate with consequent butyrogenic effect [121]. Similarly, recent studies have reported various cross-feeding interactions that exist between *Bifidobacterium* and *Bacteroides* species [122,123], and *Bifidobacterium* and *F. prausnitzii* [124]. In the same manner, other studies have reported potential syntrophic interactions between bifidobacterial strains. Of particular note are the cross-feeding activities that occur between the infant-type bifidobacterial strains *B. bifidum* PRL2010 and *B. breve* UCC2003, when these microorganisms are grown on sialyl lactose as the only carbon source [125]. Employing transcriptomic and functional genomic approaches, it was demonstrated that *B. breve* UCC2003 can cross-feed on the sialic acid released by the exosialidase activity of *B. bifidum* PRL2010 [125]. Other cross-feeding activities have been observed between a set of bifidobacterial strains such as *B. bifidum* PRL2010, *B. breve* 12L, *B. adolescentis* 22L, and *Bifidobacterium thermophilum* JCM7017, when cocultivated on plant-derived glycans such as starch and xylan [126]. Moreover, in vivo experiments using mice that had been administered *B. bifidum* PRL2010, *B. longum* subsp. *infantis* ATCC15697, *B. adolescentis* 22L, and *B. breve* 12L highlighted the existence of cross-feeding interactions between different bifidobacterial strains in the mammalian gut [127]. In fact, in these studies, the combination of transcriptomic and metagenomic approaches disclosed the evolution of the murine gut glycobiome across its enzymatic ability to degrade complex plant-derived carbohydrates such as xylo-oligosaccharides, arabinoxylan, starch, and host-glycan substrates. However, these are just some of the examples that confirm how bifidobacteria, in sharing glycan resources of the intestinal ecosystem, forge trophic relationships between intestinal microorganisms in mammals.

## 6. Bifidobacteria as Possible Microbial Biomarkers of Health Predictor

The human GIT consists of a complex and dynamic population of microorganisms, known as the gut microbiota, which have coevolved with their host [128]. The gut microbiota is considered an “essential organ” that offers many benefits to the host. In particular, gut bacteria are involved in many physiological functions such as strengthening gut integrity or shaping the intestinal epithelium [4,129], providing protection against pathogens [130,131], and influencing and regulating host immunity [4,61,132]. However, alterations of the intestinal microbiota can occur by changes in function, composition (dysbiosis), or host–microbiota interactions and they can be directly correlated with gastrointestinal diseases, such as inflammatory bowel disease (IBD), colorectal cancer, or irritable bowel syndrome (IBS) [133]. Furthermore, these alterations may be associated with other pathologies/disorders such as obesity, those affecting the respiratory tract (e.g., allergy, bronchial asthma, and cystic fibrosis) or the liver [133]. At the moment, several studies have focused on the effects and the role of bifidobacteria in those cases of dysbiosis [134,135].

In this context, the microbiota composition of necrotizing enterocolitis (NEC) patients, affected by a devastating intestinal disease most common in prematurely born infants, revealed an altered microbiota composition, in which bifidobacteria exhibit a very low abundance and prevalence during the first and second week of life, being distinctly different from a microbiota of a full-term infant [136,137]. In particular, in clinical trials that involved preterm neonates, based on the administration of *B. breve* YIT4010 and *B. breve* M-16V, separately, revealed that the treatment led to a significant reduction of infection and mortality rate [137]. These results are in accordance with Braga et al. [138], which showed that the treatment of NEC patients with the co-occurrence of *B. breve* Yakult and *Lactobacillus casei* plays an important role in reducing the incidence of this disease [137].

Furthermore, there is evidence of an alteration in the microbiota composition in other diseases such as in ulcerative colitis patients. In fact, a drastic reduction has been observed in the abundance of *B. bifidum* species in ulcerative colitis patients compared with healthy subjects [139]. Moreover, administration of *B. bifidum* PRL2010 to mice in a colitis model demonstrated a protective role in order to prevent and treat intestinal inflammation as well as re-establishment of intestinal microbiota homeostasis [139].

Studies on adenomatous polyps highlighted that abnormal tissue growth is associated with a high risk of developing colorectal cancer, based on its grade of dysplasia [135,140]. Interestingly, members of the *Bifidobacterium* genus are present in higher abundance in healthy marginal tissue when compared to colonic mucosa with polyp samples [135]. This differential bifidobacterial composition suggests the use of this genus as a biomarker for several disease conditions in biological samples [135].

## 7. Conclusions

Until today it has been widely demonstrated that bifidobacteria elicit positive effects on host health due to their metabolic and immunomodulatory activities. The study of complete bifidobacterial genomes and corresponding comparative analysis has allowed identification of various molecular mechanisms by which they interact with the host and are able to persist in the ecological niches they inhabit. The numerous benefits that have been associated with the presence of bifidobacteria, and that pertain to human physiology and the host immune system, have led to their commercial exploitation as probiotic ingredients in functional foods. Indeed, modulation of gut microbiota by means of prebiotics or probiotics could be one of the possible ways to combat intestinal disorders and improve health. However, the precise mechanism by which bifidobacteria are able to perform these important functions and are able to solicit immune responses is not fully understood. It is therefore of crucial importance that future studies fill this knowledge gap so that we will obtain a full understanding of their mode of action and allow targeted and specific applications to fully exploit their beneficial potential. Finally, despite the large knowledge that so far we have gained about bifidobacterial ecology and their genetic adaptation particularly to the human gut, much remains to be discovered about interactions and adaptations of bifidobacteria with the gut of other mammalian species.

## Figures and Tables

**Table 1:** *Bifidobacterium* (sub)species recognized as reference strains (type strains).

*Bifidobacterium* Strains	Isolation	References
*B. actinocoloniiforme* DSM 22766	Bumblebee digestive tract	[16]
*B. adolescentis* ATCC 15703	Intestine of human adult	[17]
*B. aemilianum* XV10	Carpenter bee digestive tract	[5]
*B. aerophilum* DSM 100689	Feces of cotton-top tamarin	[18]
*B. aesuclapii* DSM 26737	Feces of baby common marmoset	[19]
*B. angulatum* LMG 11039	Feces of human	[20]
*B. animalis* subsp. *animalis* LMG 10508	Feces of rat	[21]
*B. animalis* subsp. *lactis* DSM 10140	Fermented milk	[22]
*B. anseris* LMG 30189	Feces of domestic goose	[7]
*B. apri* DSM 100238	Digestive tract of wild pig	[23]
*B. aquikefiri* LMG 28769	Water kefir	[24]
*B. asteroides* LMG 10735	Hindgut of honeybee	[25]
*B. avesanii* DSM 100685	Feces of cotton-top tamarin	[18]
*B. biavatii* DSM 23969	Feces of tamarin	[26]
*B. bifidum* LMG 11041	Feces of breast-fed infant	[3]
*B. bohemicum* DSM22767	Bumblebee digestive tract	[16]
*B. bombi* DSM 19703	Bumblebee digestive tract	[27]
*B. boum* LMG 10736	Rumen of bovine	[28]
*B. breve* LMG 13208	Infant stool	[17]
*B. callimiconis* LMG 30938	Feces of Goeldi’s marmoset	[6]
*B. callitrichidarum* DSM 103152	Feces of emperor tamarin	[29]
B. callitrichos DSM 23973	Feces of common marmoset	[26]
*B. canis* DSM105923	Feces of dog	[10]
*B. castoris* LMG 30937	Feces of beaver	[6]
*B. catenulatum* LMG 11043	Adult intestine	[30]
*B. catenulatum* subsp. *kashiwanohense* DSM21854	Infant feces	[31]
*B. catulorum* DSM103154	Feces of common marmoset	[32]
*B. cebidarum* LMG31469	Feces of golden-headed tamarin	[9]
*B. choerinum* LMG 10510	Feces of piglet	[28]
*B. commune* LMG28292	Bumblebee gut	[33]
*B. coryneforme* LMG 18911	Hindgut of honeybee	[25]
*B. criceti* LMG 30188	Feces of European hamster	[7]
*B. crudilactis* LMG 23609	Raw cow milk	[34]
*B. cuniculi* LMG 10738	Feces of rabbit	[28]
*B. dentium* LMG 11045	Oral cavity	[30]
*B. dolichotidis* LMG 30941	Feces of Patagonian mara	[6]
*B. eulemuris* DSM 100216	Feces of black lemur	[35]
*B. felsineum* DSM103139	Feces of cotton-top tamarin	[11]
*B. gallicum* LMG 11596	Adult intestine	[36]
*B. goeldii* LMG 30939	Feces of Goeldi’s marmoset	[6]
*B. hapali* DSM 100202	Feces of baby common marmoset	[37]
*B. imperatoris* LMG 30297	Feces of emperor tamarin	[7]
*B. indicum* LMG 11587	Insect	[25]
*B. italicum* LMG 30187	Feces of European rabbit	[7]
*B. jacchi* DSM 103362	Feces of baby common marmoset	[38]
*B. lemurum* DSM 28807	Feces of ring-tailed lemur	[39]
*B. leontopitechi* LMG 31471	Feces of Goeldi’s monkey	[9]
*B. longum* subsp. *infantis* ATCC 15697	Intestine of infant	[17]
*B. longum* subsp. *longum* LMG 13197	Adult intestine	[17]
*B. longum* subsp. *suis* LMG 21814	Feces of pig	[40]
*B. magnum* LMG 11591	Feces of rabbit	[30]
*B. margollesii* LMG 30296	Feces of pygmy marmoset	[7]
*B. meryciucm* LMG 11341	Rumen of bovine	[41]
*B. minimum* LMG 11592	Sewage	[42]
*B. mongoliense* DSM 21395	Fermented mare’s milk	[43]
*B. moukabalense* DSM 27321	Feces of gorilla	[44]
*B. myosotis* DSM 100196	Feces of common marmoset	[37]
*B. parmae* LMG 30295	Feces of pygmy marmoset	[7]
*B. platyrrhinorum* SMA15	Feces of squirrel monkey	[45]
*B. primatium* DSM 100687	Feces of cotton-top tamarin	[11]
*B. pseudocatenulatum* LMG 10505	Infant feces	[28]
*B. pseudolongum* subsp. *globosum* LMG 11596	Rumen of bovine	[46]
*B. pseudolongum* subsp. *pseudolongum* LMG 11571	Feces of swine	[21]
*B. psychraerophilum* LMG 21775	Caecum of pig	[47]
*B. pullorum* subsp. *gallinarum* LMG 11586	Caecum of chicken	[48]
*B. pullorum* subsp. *pullorum* LMG 21816	Feces of chicken	[8]
*B. ramosum* DSM 100688	Feces of cotton-top tamarin	[18]
*B. reuteri* DSM 23975	Feces of common marmoset	[26]
*B. rousetti* BCRC 81136	Feces of Egyptian fruit bat	[49]
*B. ruminantium* LMG 21811	Rumen of bovine	[41]
*B. pullorum* subsp. *saeculare* LMG 14934	Feces of rabbit	[50]
*B. saguini* LMG 23967	Feces of tamarin	[26]
*B. saimiriisciurei* SMA1	Feces of squirrel monkey	[45]
*B. saimirii* LMG 30940	Feces of Bolivian saimiri	[6]
*B. scaligerum* DSM 103140	Feces of cotton-top tamarin	[11]
*B. scardovii* LMG 21589	Blood	[51]
*B. simiarum* DSM 103153	Feces of emperor tamarin	[11]
*B. stellenboschense* DSM 23968	Feces of tamarin	[26]
*B. subtile* LMG 11597	Sewage	[42]
*B. porcinum* LMG 21689	Feces of piglet	[52]
*B. thermacidophilum* LMG 21395	Anaerobic digester	[53]
*B. termophilum* JCM 7027	Rumen of bovine	[21]
*B. tibiigranuli* LMG 31086	Water kefir	[54]
*B. tissieri* DSM 100201	Feces of baby common marmoset	[37]
*B. tsurumiense* JCM 13495	Hamster dental plaque	[55]
*B. vansinderenii* LMG 30126	Feces of emperor tamarin	[56]
*B. vespertilionis* DSM 106025	Feces of Egyptian fruit bat	[49]
*B. xylocopae* DSM104955	Carpenter bee digestive tract	[5]
